# Engineering 3D Printed Gummies Loaded with Metformin for Paediatric Use

**DOI:** 10.3390/gels10100620

**Published:** 2024-09-26

**Authors:** Karla J. Santamaría, Brayan J. Anaya, Aikaterini Lalatsa, Patricia González-Barranco, Lucía Cantú-Cárdenas, Dolores R. Serrano

**Affiliations:** 1 Pharmaceutics and Food Technology Department, Faculty of Pharmacy, Complutense University of Madrid, 28040 Madrid, Spain; karla.santamarialpz@uanl.edu.mx (K.J.S.); branaya@ucm.es (B.J.A.); 2 School of Chemistry, Autonomous University of Nuevo León Monterrey, Monterrey 66455, Mexico; patricia.gonzalezbrn@uanl.edu.mx (P.G.-B.); lucia.cantucr@uanl.edu.mx (L.C.-C.); 3 Institute of Pharmacy and Biomedical Sciences, University of Strathclyde, Glasgow G4 0RE, UK; aikaterini.lalatsa@strath.ac.uk; 4 CRUK Formulation Unit, School of Pharmacy and Biomedical Sciences, University of Strathclyde, Glasgow G4 0RE, UK; 5 University Institute of Industrial Pharmacy, Faculty of Pharmacy, Complutense University of Madrid, 28040 Madrid, Spain

**Keywords:** 3D printing, semi-solid extrusion (SSE), 3D printed gummies, metformin, paediatrics

## Abstract

In today’s pharmaceutical landscape, there’s an urgent need to develop new drug delivery systems that are appealing and effective in ensuring therapeutic adherence, particularly among paediatric patients. The advent of 3D printing in medicine is revolutionizing this space by enabling the creation of precise, customizable, and visually appealing dosage forms. In this study, we produced 250 mg metformin paediatric gummies based on the semi-solid extrusion (SSE) 3D printing technique. A pharmaceutical ink containing metformin was successfully formulated with optimal flow properties suitable for room-temperature printing. Using a quality by design approach, 3D printing and casting methodologies were compared. The 3D-printed gummies exhibited better firmness and sustained release at earlier times to avoid metformin release in the oral cavity and ensure palatability. The texture and physical appearance match those of gummies commercially available. In conclusion, SSE allowed for the successful manufacture of 3D-printed sugar-free gummies for the treatment of diabetes mellitus for paediatric patients and is an easily translatable approach to clinical practice.

## 1. Introduction

Childhood obesity has increased exponentially in the last several decades, becoming a global healthcare issue considering that it is one of the pivotal risk factors for the development of diabetes mellitus (DM) [1,2,3,4,5]. DM belongs to the group of metabolic disorders that cause defects in glucose metabolism, resulting in a partial or whole reduction in insulin production by the pancreas [6]. Even though DM is more prominent in adults, the incidence of DM in children is becoming more frequent every day worldwide [5,7,8,9,10]. The development of type 2 diabetes mellitus (T2DM) is directly related to poor eating habits and a sedentary lifestyle. This is an alarming health issue, as T2DM progresses more aggressively in this population, causing a faster malfunction of pancreatic beta cells compared to adults [11,12]. Type 1 diabetes mellitus (T1DM) is a disease caused by the autoimmune destruction of pancreatic beta cells, thereby causing insulin deficiency throughout life. T1DM has a strong autoimmune component [13,14]. However, the increase in the body mass index (BMI) has been correlated with faster development of the disease due to the more prevalent insulin resistance in obese patients which compromises its function [8].

The recommended treatment for glucose control in pediatric patients above 10 years old diagnosed with T2DM is metformin, with an initial dose of 500 mg–2000 mg/day [15]. This drug is the first-line treatment, and if monotherapy is not effective, combination therapy with liraglutide or insulin can be used [7,8,10,15,16]. In T1DM patients, metformin is usually combined with insulin as it has been demonstrated to better control blood glucose levels, reducing the risks of hypoglycemia, and decreasing the total dose of insulin needed [17,18].

Since metformin is used for the treatment of both T1DM and T2DM, several pharmaceutical dosage forms are available in the market including immediate-release and extended-release tablets, immediate-release oral solutions, and more recently, extended-release oral suspension. However, the latter is only commercially available in a few countries [15,19]. Even though the variety of pharmaceutical dosage forms is wide, very few are paediatric appropriate formulations, which results in poor patient compliance and a lack of therapeutic adherence [7]. Consequently, the marketed metformin formulations do preclude tailored dosing and adherence due to their reduced palatability and inappropriate size for administration to a child [20]; therefore, there is a clinical need to develop paediatric appropriate dosage forms [21] able to enable a tailored dose adjusted to their needs and body weight with a suitable shape, appearance, odor, size, texture, and dosing frequency [22,23,24,25,26].

3D printing (3PD) technologies can overcome the challenges faced by the customization of the design of pediatric pharmaceutical dosage forms [27,28]. Some of the 3DP technologies allow a layer-by-layer ink deposition until the desired shape is obtained [29]. Nowadays, this technology has gained interest within the pharmaceutical industry to produce tailored medications adjusted to patients’ needs [27]. The feasibility of 3DP polypills for metabolic syndrome has been previously demonstrated [30]. However, solid dosage forms are not the ideal formulation for children as they prefer chewable formulations, such as medicinal gummies; appropriate organoleptic characteristics target better therapeutic adherence and compliance and reduce the psychological impact of the disease [31,32,33,34,35].

There are several 3DP technologies implemented in the fabrication of solid dosage forms [36]. These are mainly differentiated according to the type of material that is deposited layer by layer, as well as the final characteristics of the dosage forms. For example, within the material extrusion techniques, fused deposition modeling (FDM) relies on thermoplastics for extrusion and adhesion with the previous layers [37]. Even though this is one of the most popular techniques due to its low cost and speed [38], its main disadvantage is the high temperatures required to go above the glass transition temperature of the thermoplastic, which limits its use with thermosensitive drugs [39].

Another technique used is vat polymerization, where a liquid photosensitive resin is solidified by photopolymerization, using ultraviolet light to start the process and form a solid structure [37,39]. This technique stands out for the high resolution of the products obtained, although its main limitation lies in the materials used since the specifications and identification of risks are not yet clearly defined especially for the paediatric population [40].

On the other hand, binder jetting is a technique in which the material is a powder, and the particles adhere to each other by means of a binder that is deposited from head-on between successive layers of powder. However, the final product characteristics are very far from those required for a gummy [37].

Amongst all 3DP techniques, semi-solid extrusion (SSE) has shown better performance in producing medicinal gummies [33,41,42]. Patient-tailored medicinal gummies or ‘drugmies’ are dosage forms with eye-catching appearances and appropriate organoleptic characteristics that can improve treatment adherence and reduce the psychological impact of the disease, especially in children. Gelatin, the main component of gummies, is an ideal hydrocolloid for forming hydrogels, which facilitates its use in the formulation of semi-solid gels used in SSE. The basis of this technique lies in the sequential deposition of material layer by layer, using gels with suitable rheological properties, until a 3D-printed gummy is obtained [43]. One of the main advantages of this technique is the low temperature required for printing, which allows for working with thermosensitive active ingredients [43]. Nevertheless, one of the main challenges of SSE is to optimize the rheological behavior of the ink [44], as the printability of the ink and the final mechanical strength of the product directly depends on the rheological properties, including storage modulus, loss modulus, and complex viscosity [45].

The hypothesis underpinning this work is that semisolid metformin gel formulations with balanced rheological properties could be utilized to produce tailored 3D-printed gummies able to adjust to the required paediatric dose for the treatment of DM. Conventional casting technologies will be compared with 3DP SSE able to optimize metformin-loaded gummies to treat T2DM/T1DM using a quality by design approach. A full physicochemical characterization and the dissolution profile of the optimal 3DP metformin gummies were undertaken to understand their potential translation for paediatric use.

## 2. Results and Discussion

### 2.1. Design of Experiments (DoE)

#### 2.1.1. Quality by Desing (QbD)-Based Model Development and Response Surface Analysis

A full two-level DoE analysis was conducted using a multi-linear regression analysis method. The coefficients of the model equations generated for each Critical Quality Attribute (CQA) revealed the goodness of fit of the experimental data to the selected model with a *p*-value < 0.05 related only to the type of process employed (3D printing or casting). High values of R^2^ = 0.8751 and R^2^ = 0.9870 for firmness and drug release were obtained respectively.

**Figure 1:**
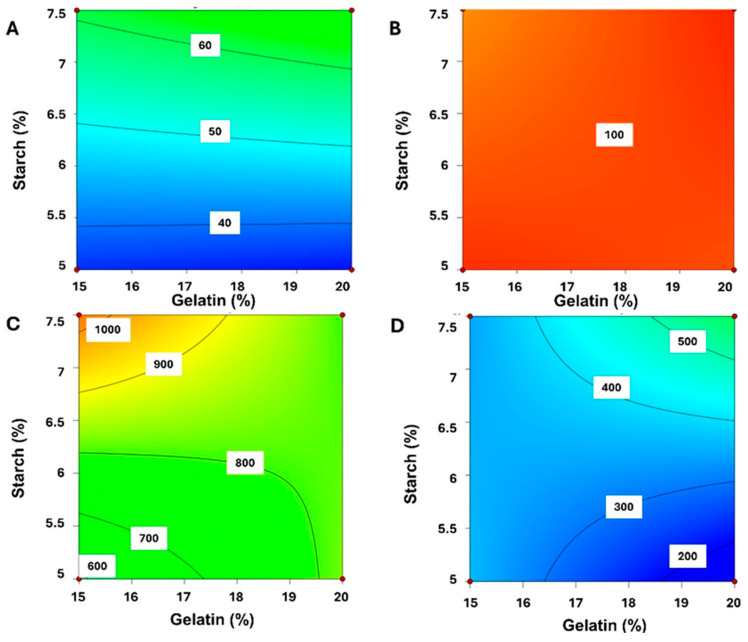
2D Contour plots for optimization of MET gummies. Key: (**A**,**B**) Durg Release; (**C**,**D**) Firmness. The effect of 3D printing on firmness and drug release is represented in (**A**,**C**) while the effect of casting is illustrated in (**B**,**D**).

Figure 1 presents the 2D contour plots identifying the relationship between the percentage of starch and gelatin in the 3D printing process (A,C) compared to the casting fabrication (B,D). The percentage of starch played a key role in the dissolution profile when gummies were obtained by 3DP while no effect was observed for casted gummies as all exhibited a fast drug release close to 100% in 5 min. The larger the amount of starch in the formulation, the faster the dissolution in aqueous media for 3DP gummies (Figure 1A,C). Nevertheless, the amount of gelatin did not impact significantly on the dissolution of the 3D-printed gummy (Figure 1B). Regarding the firmness of the gummies, an antagonistic effect was observed for the gelatin between the casting and 3D printing methods (Figure 1C,D). The firmness increased when a greater amount of starch was used for both methodologies, but the percentage of gelatin impacted differently. For 3DP gummies, the lower the gelatin, the better the firmness, while for casted gummies, the higher the gelatin, the greater the firmness.

#### 2.1.2. Optimal Formulation and Validation of QbD

The optimal formulation was defined through the exchange of several CQAs to obtain the desired objectives prioritizing the sustained drug release and the enhancement of the gummy’s firmness. Considering the above, the optimized formulation consisted of 20% gelatin, 5% starch, and 3DP as the manufacturing process. The results obtained showed a 77% drug release at five minutes with a firmness equivalent to 6.5 N. The validation of the mathematical modeling revealed that the firmness values agreed with the predicted ones within the 95% confidence interval [5.2 N–10.9 N]. However, the drug release prediction deviated from the experimental values [12.5–55.5%], exhibiting a faster release profile.

### 2.2. Optimization of Gummies

#### 2.2.1. Casting Method

**Figure 2:**
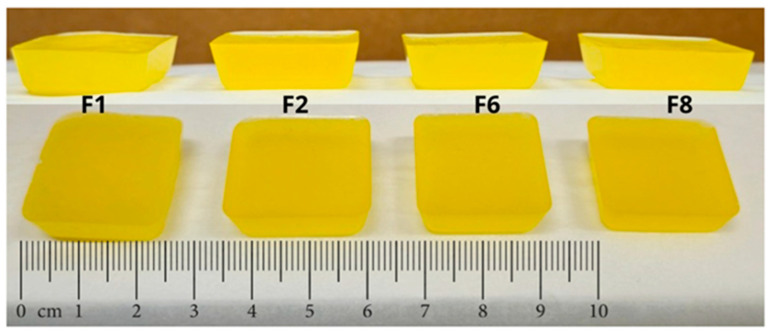
Appearance and size of gummies cast. Composition is described in Table 3 in the methodology section.

Gummies prepared by the casting (Formulations F1, F2, F6, and F8) presented adequate characteristics after the cooling time to facilitate the removal of the gummies from the mold without any loss or deformation. The gummies had the following dimensions: 24.5 mm × 24.5 mm × 7.1 mm with a final weight of 4.2 ± 2.12 g presenting a smooth and homogeneous surface, and a yellow color attributed to the essential oil of orange peel (Figure 2, Table 1).

#### 2.2.2. 3D Printing SSE

**Figure 3:**
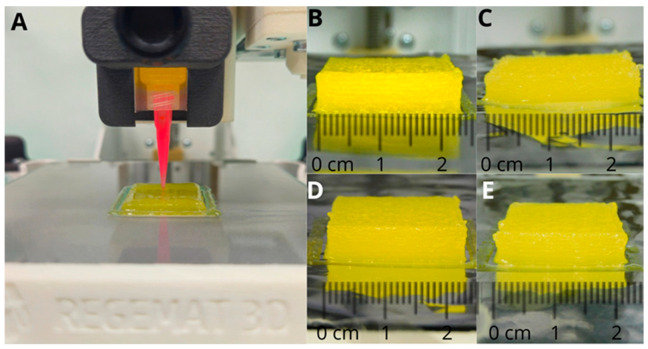
Appearance and size of gummies prepared using 3DP SSE. (**A**) Printing process; (**B**) F3; (**C**) F4; (**D**) F5; and (**E**) F7. Composition is described in Table 3 in the methodology section.

The preparation of gummies by 3DP SSE (Formulations F3, F4, F5, and F7) was more challenging. The printing process was carried out immediately after the ink preparation to ensure appropriate flow. Otherwise, the viscosity of the ink increased, hampering the correct deposition on the platform after 30 min. Formulations F3 and F5 showed better deposition characteristics compared to F4 and F7 in which the ink tended to solidify faster, disrupting the surface of the gummy. F3 and F5 formulations exhibited a smooth and homogeneous surface devoid of particles, stains, or heterogeneously colored regions (Figure 3), thereby confirming the suitability of the ink and printing parameters.

The 3D-printed material was deposited in successive layers on aluminum foil. After cooling, the gummies were readily removed from the foil, without any loss or deformation. The resulting gummies exhibited visually and tactile appealing characteristics (Figure 3). However, the weight variability was higher than with the casting method (3.7 ± 0.7 g) (Table 2).

### 2.3. Content Uniformity and Mass Uniformity

The formulations were designed to contain 250 mg of MET. The optimized 3D-printed gummies had a content uniformity of 92.84% ± 2.35% with a 2.53% RSD and a mass uniformity of 3.79 g ± 0.18 g (4.91% RSD).

### 2.4. Physicochemical Characterization and Organoleptic Properties

#### 2.4.1. Powder X-ray Diffraction

**Figure 4:**
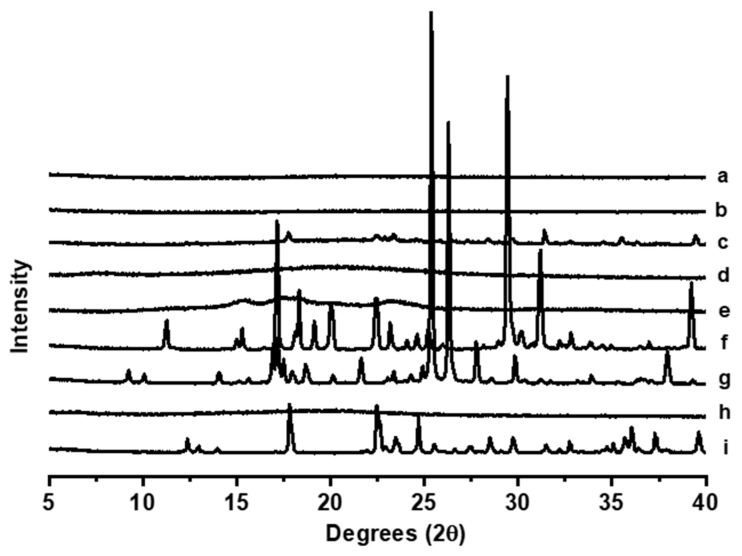
PXRD analysis. Key: (**a**) Optimized 3D-printed gummy without MET, (**b**) Optimized 3D-printed gummy within MET, (**c**) Physical mixture, (**d**) Gelatine, (**e**) Wheat starch, (**f**) Citric acid, (**g**) Nipagin (Methyl Paraben), (**h**) Xanthan gum, and (**i**) Unprocessed MET.

The pXRD analysis of the excipients, MET, and the optimized 3DP formulation with and without MET is shown in Figure 4. Unprocessed MET exhibited characteristic Bragg peaks that were shown in the physical mixture among all the components. However, after the 3D printing process, gummies with and without MET exhibited a characteristic amorphous halo indicating that the drug did not crystallize upon printing and cooling, which could result in poorer palatability.

#### 2.4.2. DSC-TGA Analysis

**Figure 5:**
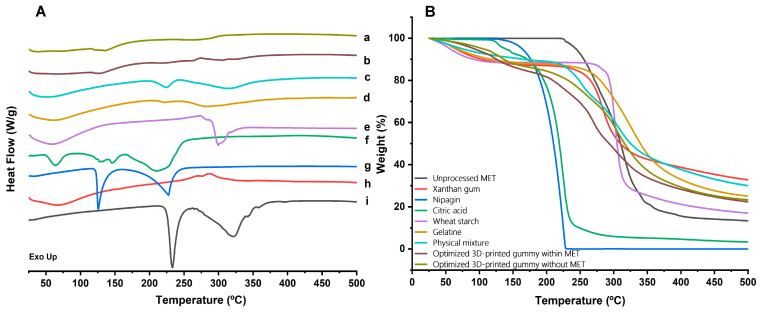
(**A**) DSC and (**B**) TGA analysis. Key: (**a**) Optimized 3D-printed gummy without MET, (**b**) Optimized 3D-printed gummy within MET, (**c**) Physical mixture, (**d**) Gelatine, (**e**) Wheat starch, (**f**) Citric acid, (**g**) Nipagin (Methyl Paraben), (**h**) Xanthan gum, and (**i**) Unprocessed MET. A colour legend is also facilitated within the figure for clarity.

The DSC-TGA analysis reveals distinct thermal profiles for the 3D-printed gummies and unprocessed materials (Figure 5). The gummies exhibited fewer and broader endothermic peaks compared to individual components, indicating successful ingredient miscibility and an amorphous structure. Key endothermic events include the onset of melting for citric acid and metformin at 153 and 233 °C respectively. The absence of the MET melting event was found in the 3D-printed gummies, while the melting event attributed to citric acid was still present. The TGA curves showed improved thermal stability for the 3D-printed gummy until 150 °C compared to the raw materials, followed by a significant weight loss occurring above 200 °C. The 3D-printed gummy without MET showed better thermal stability as well as physical mixture, which can be attributed to the presence of MET.

#### 2.4.3. Scanning Electron Microscopy (SEM)

**Figure 6:**
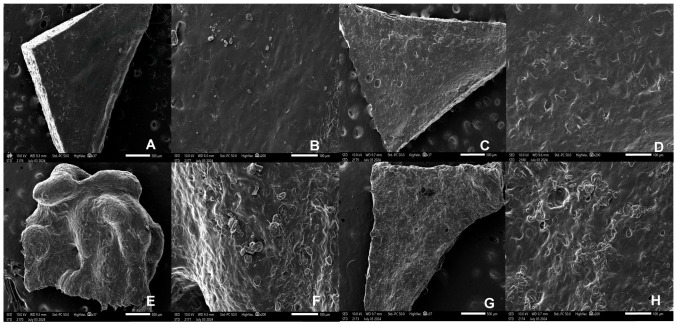
SEM micrographs of 3D printed gummies with and without MET at different magnifications. (**A**) 3D printed gummy surface with MET 37× (500 µm); (**B**) 3D printed gummy surface with MET 200× (100 µm); (**C**) 3D printed gummy middle layer with MET 37× (500 µm); (**D**) 3D printed gummy middle layer with MET 200× (100 µm); (**E**) 3D printed gummy surface without MET 37× (500 µm); (**F**) 3D printed gummy surface without MET 200× (100 µm); (**G**) 3D printed gummy middle layer without MET 37× (500 µm); (**H**) 3D printed gummy middle layer without MET 200× (100 µm).

In the SEM micrographs, the surface and intermediate layers of the 3DP gummies with and without MET are illustrated (Figure 6). A homogenous ink deposition and smooth surface were observed in the 3DP gummy containing MET as well as in the middle layer, indicating a robust 3D printing process after optimization. However, unloaded gummies without MET exhibited a rough surface, indicating a poorer miscibility of components. The presence of voids was also observed which can be attributed to more deficient ink deposition. The same microstructure was visualized in the intermediate layers.

#### 2.4.4. Organoleptic Properties

All the gummies showed a homogeneous yellow-orangish colour with an orange smell due to the addition of orange essential oil. The shape was well-defined in all cases, both for the casted and printed gummies. According to the tactility scale, cast gummies exhibited a soft and slightly chewy behaviour while 3D printed gummies were moderately firm and chewy.

### 2.5. Mechanical Strength

**Figure 7:**
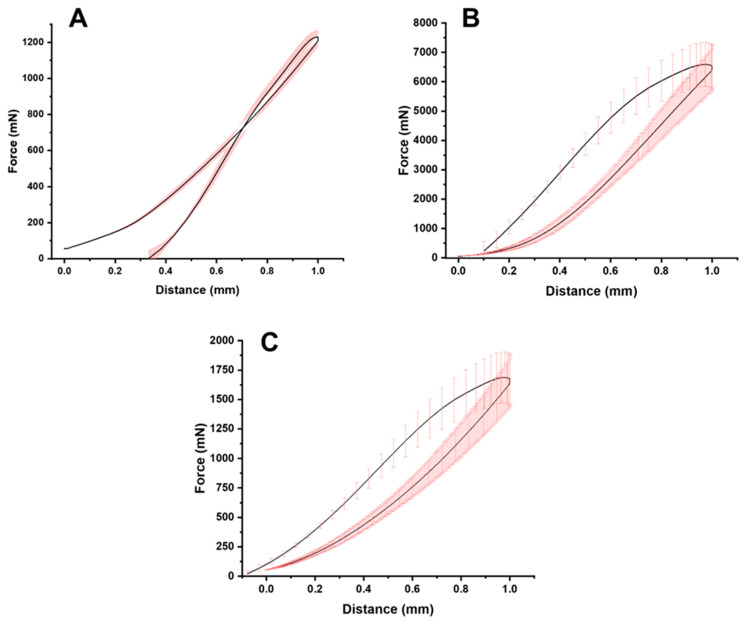
Texture analysis evaluation (*n* = 3). Keys: (**A**) Commercial gummy, (**B**) 3D printed gummy after 24 h post-processing, and (**C**) 3D printed gummy after 72 h post-processing.

Figure 7 illustrates the mechanical compression of the gummies. The maximum recorded force exerted by each gummy was 1229.6 ± 36.4 mN, 6589.3 ± 749.1 mN, and 1687.8 ± 216.9 mN for the commercial gummy, 3D-printed gummy after 24 h, and 3D-printed gummy after 72 h respectively, while the area under the curve was 526.2 ± 19.5 mN·s, 2556.9 ± 237.2 mN·s, and 717.3 ± 85.9 mN·s respectively. It is worth noting that the firmness of the 3D-printed gummy was reduced over time reaching similar values between commercial gummies and 3DP gummies after 72 h post-processing.

### 2.6. Rheological Evaluation

**Figure 8:**
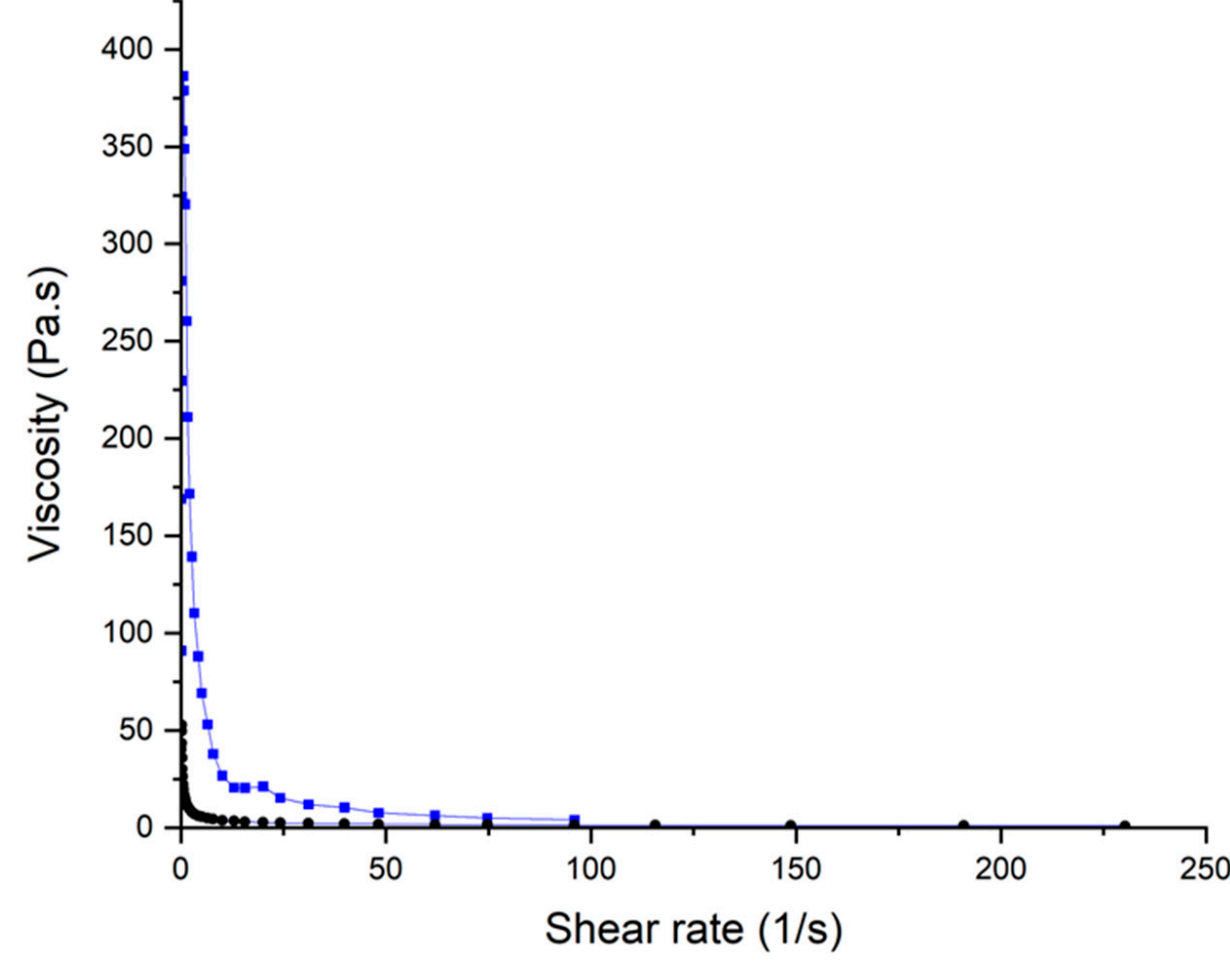
Rheological behavior of the gel used for 3D printing. Key: 3D printed gummies with MET (-▪-), 3D printed gummies without MET (-●-).

Rheological analysis revealed a significant impact due to the incorporation of MET in the gel ink (Figure 8). Both inks, with and without MET, showed a characteristic shear-thinning behavior in which the viscosity decreases upon an increase in the shear rate. However, the gel ink containing MET showed a superior viscosity at very low shear rates which facilitates keeping the shape after printing. Also, a drastic drop in the viscosity was observed in the ink containing MET even when low shear rates were applied. This is indicative of a suitable flow from the syringe during printing which can explain the smoother surface of the gummies containing MET.

### 2.7. Dissolution Profile

**Figure 9:**
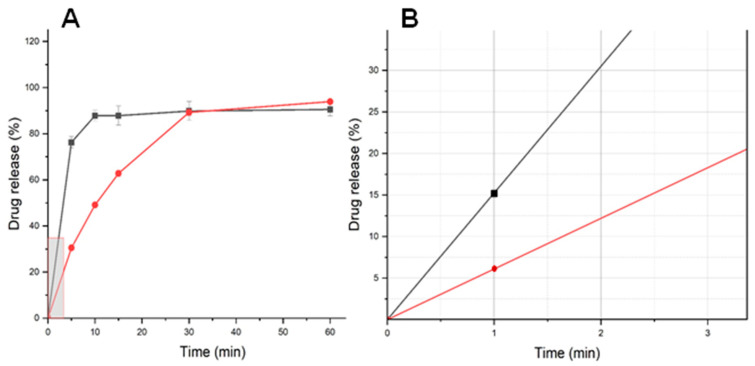
(**A**) Dissolution profile of optimized 3D-printed gummy; (**B**) Enlargement of the dissolution profile at earlier time points. Keys: optimized 3D-printed gummy (-■-), to commercial MET tablet (-●-).

The dissolution profile of 3DP gummies compared to commercial tablets is illustrated in Figure 9. For a gummy, immediate release can reduce palatability so release should occur at least after a few minutes (>2–3 min), being likely to have better adherence. The dissolution profile of the 3D-printed gummy showed a significantly faster release compared to the commercial MET tablet (*p*-value < 0.05). However, as illustrated in Figure 9B, the percentage released at 1 min, the time required to chew and swallow the gummy, was 15% which when combined with suitable flavoring agents to mask the bitter taste of MET is unlikely to lead to bitterness that will limit compliance. 

3D-printed gummies are personalized medicines that can enable the tailoring of doses for paediatric populations. This approach enables precise control over dosage, composition, and geometry, potentially enhancing therapeutic efficacy and patient compliance. While challenges in achieving consistent drug release profiles and meeting regulatory standards persist, ongoing research is rapidly addressing these issues. Optimization of printing parameters, development of advanced dissolution testing methods, and careful selection of printing techniques are key focus areas. As these challenges are overcome, 3D-printed gummies are poised to significantly improve pediatric pharmacotherapy, offering a new paradigm in personalized medicine that could markedly enhance treatment outcomes and patient experience.

To the best of our knowledge, for the first time, the feasibility of manufacturing 3D-printed gummies containing MET has been demonstrated for the treatment of T1DM and T2DM for the pediatric population as a friendlier and more attractive dosage form [46]. Developing a gummy formulation containing MET is challenging due to the need for precise dosing, high dose, stability of the active ingredient, and the potential for flavorings or other ingredients in gummies that may interact negatively with the medication, or the condition being treated. The use of 3D printers can facilitate the clinical translation of tailored gummies from the hospital to patients. In terms of printability, the flow velocity of the ink was 2 mm/s and the cross-sectional area of the nozzle was 0.264 mm^2^ resulting in a volumetric flow rate of 0.528 mm^3^/s that was calculated using the following equation:

*Q* = *v* × *A* (1)

where *v* is the flow velocity expressed in mm/s and A is the cross-sectional area of the nozzle expressed in mm^2^. Knowing the volumetric flow rate, it is possible to estimate the shear rate during extrusion using Equation (2):

(2) $$ϒ=\frac{4Q}{\Pi r^{3}}$$

where *Q* is the volumetric flow rate (extrusion speed during printing expressed in mm^3^/s), and r is the radius of the nozzle. The estimated shear stress during printing was 28 s^−1^.

It is worth highlighting the importance of developing a suitable ink for SEE purposes. When preparing inks used for SSE printing, viscosity is a crucial factor [47]. Ideal viscosity typically falls within 30–10,000 Pa·s, depending on the balance between extrusion ease and post-print stability. In our case, the ink prepared exhibited a low viscosity, behaving as a fluid-like material at 30 Pa·s with shear rates relevant to extrusion (28 s^−1^). This ink was easier to extrude but requires gelation to maintain shape after printing, which ideally should be increased when more complex geometries are printed.

Therefore, it should be considered that the viscosity of a material is adequate if it is low enough during extrusion so that it can flow through a nozzle, but enough to support and maintain the structure deposited layer by layer [35,48]. The inks prepared in this study contained the appropriate ratio of gelatinizing agents that, when interacting with MET, presented a higher viscosity that is necessary to allow the process to be carried out at room temperature, without the need to heat the tip or cool the printing base [34,49]. One of the reasons behind this evidence can be due to the multiple H-bond donor groups that the MET molecule possesses, which are likely to form weak physical interactions with the excipients that upon increase in the shear rate will break, allowing for an easy flow, but after deposition on the platform can be re-established again. This also justified the high firmness found in the 3D-printed gummy compared with other commercial gummies mostly containing gelling agents. However, a post-processing step based on refrigerated storage resulted in a loss of firmness like commercial gummies, which can be attributed to the reduced molecular mobility at lower temperatures, harnessing physical interactions.

The physicochemical characteristics, as well as the physical appearance of the 3D gummies, are key to guaranteeing patient adherence. The optimized gummy presented a visual and tactile appearance that did not differ from the commercial gummies. A homogeneous color, particles or lumps not visible to the naked eye, a smooth surface, and an attractive aroma were achieved. Gelatin concentration plays a crucial role in determining the mechanical strength and dissolution rate of gummy formulations. Regarding mechanical strength, we observed that gummies printed with lower gelatin concentrations exhibited increased firmness. This effect is attributed to the renaturation process of the gelatin, in which the polymer chains rearrange to their original configuration as the temperature drops from 40 °C to room temperature and then post-refrigerated conditions. During extrusion, the shear forces applied as the material passes through the nozzle facilitate this molecular rearrangement, enabling the process to occur even at low gelatin concentration [50].

The dissolution kinetics of 3D-printed gummies were significantly influenced by formulation composition and printing parameters. Our dissolution studies demonstrated that the novel 3D-printed gummy formulation exhibited drug release profiles comparable to those reported in recent literature for pediatric gummy formulations based on gelatin and HPMC hydrogels [35]. Both our formulation and those previously reported displayed rapid dissolution characteristics, with approximately 85% drug release occurring within 15 min. The faster drug release from 3D-printed gummies can result in a quicker onset of action, eliciting better control of the post-prandial glycaemia in children compared to commercially available MET tablets that exhibit a 50–60% oral bioavailability with a t_max_ at 2.5 h [51].

On the other hand, a feature worth highlighting is that drug release at earlier times (<1 min) was below 20%, allowing to swallow the gummy while preventing MET release and maintaining suitable palatability. However, further in vivo studies are required to confirm efficacy, as well as organoleptic testing to verify the acceptability of these gummies to paediatric patients. Stability testing of the gummies has not been undertaken, but as this medicine is intended for personalized dosing, these studies will only provide information for increasing the cost-effectiveness of the approach and limited stability studies (over 7–28 days) could support first-in-human studies. This research marks a significant advance in the development of effective and patient-friendly treatment options for managing diabetes in children.

Compared to other 3D-printed gummies in the literature, the release control of the active ingredient was achieved by using just 5% starch combined with 3.3% active ingredient [34]. However, in our study, the amount of MET included was almost double (6.25%) while using the same percentage of starch. MET has many ionizable functional groups at acidic pH which makes the sustained release difficult upon disintegration. However, it is expected that increasing the ratio of starch within the formulation would lead to stronger interactions and a prolonged MET release. Additionally, the gelatinization process is also crucial for the development of this type of formulation. During the gelling process heat was required, but this can promote the destruction of the crystalline structure of the starch [52], hampering the interactions with the active ingredient.

## 3. Conclusions

This study has successfully demonstrated the feasibility of utilizing 3D printing SSE technology to create a novel gummy dosage form containing MET, tailored specifically for the pediatric population with T1DM and T2DM. The optimized formulation not only addresses the challenges associated with precise dosing and stability during printing but also achieves a palatable and visually appealing product, which is crucial for patient adherence. The integration of appropriate gelatinizing agents and careful consideration of viscosity during the SSE printing process ensured the structural integrity and desirable drug release profile of the 3D-printed gummies. Importantly, our findings suggest that this innovative dosage form could offer a faster onset of action compared to traditional MET tablets, potentially improving post-prandial glycemia control in children. However, further in vivo studies are required to confirm efficacy, as well as organoleptic testing to verify the acceptability of these gummies to pediatric patients. Stability testing of the gummy over time is also needed, and the potential to optimize the release profile through formulation adjustments is explored. This research marks a significant advance in the development of effective and patient-friendly treatment options for managing diabetes in children.

## 4. Materials and Methods

### 4.1. Materials

Metformin hydrochloride (MET) (purity 99.89%, Ph. Eur) was a gift from Globe Chemicals (México City, México). Wheat starch was purchased from Guinama (Madrid, Spain). Xanthan gum was purchased from MP Biomedicals (Madrid, Spain). Citric acid monohydrate (99.5% purity) was purchased from Thermo Scientific (Madrid, Spain). Nipagin (methyl paraben) was purchased from Fagron (Madrid, Spain). Gelatin (Royal, Mondeléz, Madrid, Spain Commercial, S.L.), agar-agar (Vahiné, McCormik España S.A.), liquid sweetener (Steviat, SoriaNatural, Madrid, Spain), glycerin (Azucren, Artynnova reposteria Sevilla), and orange essential oil (Herbolario Navarro España) were bought from a local store (Madrid, Spain). Purified water was obtained through an Elix 3, Millipore purified water system (Merck, Madrid, Spain). The HPLC-grade solvents were purchased from Proquinorte (Madrid, Spain). All other reagents were used without further purification and were of analytical grade.

### 4.2. Quality by Design Approach

A quality by design approach was utilized to find the optimal ratio between excipients and the most appropriate manufacturing method. A simple 2^3^ factorial design was performed using Design-Expert 13 software (Stat-Ease Inc, Minneapolis, MN, USA). The objective of this DoE was to evaluate how the two main excipients (gelatin and starch) and the manufacturing method (casting and 3DP) influence the firmness and drug release from a gummy loaded with MET.

The amount of MET (6.25% *w*/*w*), citric acid (0.5% *w*/*w*), nipagin (0.125% *w*/*w*), orange essential oil (3 drops), glycerin (3 drops), sweetening liquid (2 drops), and water (4 mL) were kept constant while gelatin, agar-agar, starch, and xanthan gum used as gelling agents were varied according to the DoE matrix to complete 100% of the total weight of the gummy (Table 1).

The following factors were investigated at two levels: (i) the percentage of gelatin (15% or 20%), (ii) the percentage of starch (5% or 7.5%), and (iii) the manufacturing method (casting or 3D printing), described in Table 3. The remaining components were adjusted with agar-agar and xanthan gum to add up to 100% of the total weight. Formulation optimization was performed to maximize consistency and foster a sustained MET release.

### 4.3. Gummy Preparation

#### 4.3.1. Casting Method

The four formulations corresponding to the casting method were prepared according to the DoE (Table 1). The composition of these formulations differed mainly in the percentages of gelatin and starch, as well as the presence or absence of agar-agar and xanthan gum needed to bulk it up to 100%. In addition, excipients, including sweeteners and essential oils, were used to improve the organoleptic properties and palatability of the gummies.

First, MET, citric acid, and nipagin were dissolved in 2 mL of purified and heated water (75 °C), maintaining gentle and constant stirring throughout the process. Then the starch was incorporated and once a uniform mixture was achieved, the remaining 2 mL of water was added. Then, gelling agents, such as gelatin, xanthan gum, and agar-agar were added individually, allowing enough time for full hydration. Finally, the orange essential oil, the sweetener, and the glycerin were incorporated. The formulation was then placed into a 24 mm × 24 mm square mold and stored in a refrigerator at 4 °C for 12 h.

#### 4.3.2. 3D Printing Semisolid Extrusion

The same procedure previously described for the casting method was employed for the preparation of the ink. Subsequently, 5 cc plastic syringes with a 0.58 mm diameter dispensing tip were filled with the inks and were loaded directly into the bioprinter (REG4LIFE 3D bioprinter, REGEMAT 3D, Granada, Spain). The same printing design was created directly in the Regemant Software and consisted of a 24 mm × 24 mm cube with a 6 mm height. The printing layer height was set to 0.40 mm. The printing process was performed at room temperature with a flow rate of 2.00 mm/s and a solid infill pattern with a 90° angle. The total printing time was approximately 32 min.

#### 4.3.3. Search for Optimum Formulation and Validation Studies

Mathematical modeling was performed using multiple linear regression analysis (MLRA). In constructing the polynomial equations, only statistically significant coefficients (*p* < 0.05) were included. The model’s performance was assessed by examining the *p*-value, and the coefficient of determination. To explore the relationships between various factors and responses, response surface analysis was conducted using 2D contour plots. Optimal formulation prediction was carried out through numerical optimization and desirability functions, targeting the greatest firmness of the gummy and the lower drug release at 5 min in aqueous media. MET has a bitter taste which should be hindered for several minutes to enhance patient compliance. The QbD methodology was validated by comparing predicted responses with observed data using linear correlation and residual plots [53].

### 4.4. Content Uniformity and Mass Uniformity

The formulations were designed to contain 250 mg of MET. The optimized 3D-printed gummies (*n* = 5) were weighed on an analytical balance and then dissolved in 100 mL of deionized water. Content uniformity was quantified by HPLC using the method below described.

### 4.5. Physicochemical Characterization

The solid-state characterization was conducted using the optimized 3D-printed gummy formulation with MET and without MET, the excipients, and the unprocessed MET powder. A physical mixture with the same drug/excipients ratio was prepared in an agate mortar and pestle. The studies were performed at the CAI Technology Research Center (Centro de Asistencia a la Investigación, UCM, Madrid, Spain).

#### 4.5.1. Powder X-ray Diffraction

A powder X-ray diffraction analysis was conducted on each of the raw excipients, raw MET, the physical mixture of powders, and the optimized printed gummy with and without MET. A Philips^®^ X’Pert-MPD X-ray diffractometer (Malvern Panalytical^®^; Almelo, The Netherlands), equipped with Ni-filtered Cu K radiation (1.54), was used to perform the powder X-ray analysis. A voltage of 40 kV and a current of 40 mA were employed to conduct the study. PXRD patterns were recorded at a step scan rate of 0.05° per second from 5° to 40° on the 2-thetas scale [30]. For comparison purposes, physical mixtures of raw powder materials between API and excipients, prepared in an agate mortar and pestle were used.

#### 4.5.2. Differential Scanning Calorimetry

DSC-TGA Standard scans were performed using nitrogen as the purge gas on an SDT Q600 V8.3 instrument (TA instruments, Elstree, UK) calorimeter. Samples were left to dry at room temperature for 72 h before measurement took place. Analysis was performed at a scanning rate of 10 °C/min from 25 °C to 500 °C. The instrument was calibrated using indium as the standard. The glass transition temperatures reported are the midpoint of the transition (*n* = 3).

#### 4.5.3. Scanning Electron Microscopy

The morphological characteristics of the optimized 3D-printed gummy, loaded with MET, and an optimized 3D-printed gummy without MET were evaluated. Slices obtained from the surface and the middle part of the gummy were cut and placed onto 32 mm stubs to dry before measurements. Samples were coated with pure gold for 180 s (Q150RS QUORUM Metallizer, Madrid, Spain). A scanning electron microscope (JSM-IT700HR JEOL, Tokyo, Japan) at 10 kV was used.

#### 4.5.4. Organoleptic Evaluation

The organoleptic properties of the gummies were visually assessed focusing on color homogeneity, shape, and smell. To describe the tactility of gummies, the following 5-point scale was applied:

1. Soft and squishy: Extremely soft to the touch, yielding easily with little pressure, minimal resistance when biting. Tends to dissolve quickly in the mouth.
2. Soft and slightly chewy: Soft but with a little resistance when pressed. Offers a more satisfying bite but is still easy to chew and dissolves relatively quickly.
3. Moderately firm and chewy: Balanced between firmness and softness. Requires noticeable effort to bite and chew, offering a consistent chew throughout.
4. Firm and very chewy: Solid texture with significant resistance when biting. Takes longer to chew and breaks down more slowly.
5. Hard and sticky: Very firm to the touch and chewy with a sticky texture. Difficult to bite into and sticks to teeth when chewing.

### 4.6. Mechanical Strength

The compression strength of the MET-loaded gummies formulation was evaluated in triplicate using the Texture Analyzer TA.XT Plus C (Stable Micro Systems Ltd., Godalming, UK). The test was applied for the 3D printed gummy after 24 h and 72 h post-processing, and results were compared with a commercial gummy (Haribo, Getafe, Spain). The force required to compress the 3DP gummy was determined. The gummy was mounted onto the center of the base of the texture analyzer. A cylindrical probe with a diameter of 25 mm (p/25 P) then made contact with the gummy at a constant speed of 0.5 mm/s. Once the probe was in contact with the gummy, the force exerted to displace the probe down by 1 mm was recorded. Afterward, the probe was detached at a post-test speed of 10 mm/s. Data was collected at a rate of 200 points per second (PPS). The area under the curve (AUC) from the force-distance plot was used to quantify the compressibility (firmness) of the 3DP gummies using the Exponent software (version 8.0.14.0, Stable Micro Systems, Godalming, UK).

### 4.7. Rheology

Rheological characteristics were conducted in triplicate using an AR2000 Rheometer (TA Instruments, Newcastle, DE, USA) and a 40 mm flat plate geometry. The rheology was tested following the evolution of viscosity versus shear rate. The rheometer was configured to increase the shear rate by 0.33 Pa/s up to 75 s^−1^. The collected data were analyzed using TA Universal Analysis software (Waters, New Castle, DE, USA).

### 4.8. Dissolution Profile

To evaluate the release profile of the optimized 3D-printed gummy, dissolution tests were performed in triplicate. Full square shape printed gummies were tested. Other more complex geometries were not evaluated as the consistency of the ink post-printing did not allow to maintain the morphology. The apparatus employed was the United States Pharmacopeia (USP) II apparatus (ERWEKA DT 80, Heusenstamm, Germany). A stirring speed of 100 rpm was employed to investigate the drug release profile under sink conditions, following the USP 2021 guidelines. The dissolution medium utilized was a simulated gastric fluid (SGF) (HCl 0.1 N at pH 1.2) prepared according to the United States Pharmacopeia (USP) standards, without enzymes [54]. Samples (1.5 mL) were collected at different times: 1 min, 5 min, 10 min, 15 min, 30 min, and 60 min, and then were filtered through a 0.45 µm hydrophilic filter (Millipore, Millex-LCR, Madrid, Spain). For purposes of comparison, the dissolution profile of 850 mg commercial MET tablets (Laboratorios Cinfa, Navarra, Spain) was also studied.

#### Quantification of MET by High-Performance Liquid Chromatography (HPLC)

Samples were diluted 1:10 with deionized water. HPLC analysis was performed using a Varian Prostar 230 solvent delivery module, a Varian Prostar 410 autosampler, and a Varian Prostar 310 UV-visible detector (Varian, Palo Alto, CA, USA). Peak integration was performed with a Galaxie chromatography data system (Varian, CA, USA). MET was eluted on a C18 THERMO Scientific Betasil phenyl DIM (mm) 150 mm × 4.6 mm, using a 5 µm internal particle size column. The mobile phase consisted of methanol: water (78:22 *v*/*v*) pumped at 0.3 mL/min. The sample injection volume was 20 µL, and the detector was set at 236 nm [55]. A calibration curve between 6 µg/mL and 64 µg/mL was formed. A good correlation between drug concentration and absorbance was obtained within this range (R^2^ = 0.9996).

### 4.9. Statistical Analysis

Statistical analysis (two-sample *t*-test) for dissolution data was performed using Minitab v.19 (Minitab Ltd., Coventry, UK). Graphs were plotted with the use of Origin 2021 (OriginLab Corporation, Northampton, MA, USA).

## Figures and Tables

**Table 1:** Dimensions and weight of gummies using the casting method.

Formulations	Height (mm)	Width (mm)	Length (mm)	Weight (g)
F1	7.4	24.5	24.7	4.3193
F2	7.3	24.7	24.5	4.1445
F6	7.1	24.9	24.5	4.2605
F8	6.9	24.5	24.7	4.2318

**Table 2:** Dimensions and weight of gummies using 3DP SSE.

Formulations	Height (mm)	Width (mm)	Length (mm)	Weight (g)
F3	6.1	23.8	24.0	4.1217
F4	4.3	24.0	24.3	2.6848
F5	5.7	24.1	23.8	4.0355
F7	5.9	24.5	24.1	3.9868

**Table 3:** DoE Matrix factors and levels.

	Gelatin (%)	Starch (%)	Process
F1	15	5.0	Casting
F2	20	7.5	Casting
F3	15	5.0	3DP
F4	15	7.5	3DP
F5	20	5.0	3DP
F6	20	5.0	Casting
F7	20	7.5	3DP
F8	15	7.5	Casting

## Data Availability

Data will be made available upon request.

## References

[1] Xu H., Verre M.C. (2018). Type 2 Diabetes Mellitus in Children. Am. Fam. Physician..

[2] Di Cicco M., Ghezzi M., Kantar A., Song W.J., Bush A., Peroni D., D’Auria E. (2023). Pediatric obesity and severe asthma: Targeting pathways driving inflammation. Pharmacol. Res..

[3] Argelich E., Alemany M.E., Amengual-Miralles B., Argüelles R., Bandiera D., Barceló M.A., Beinbrech B., Bouzas C., Capel P., Lònia Cerdà A. (2021). Paediatric teams in front of childhood obesity: A qualitative study within the STOP project. An. Pediatr..

[4] Lasarte-Velillas J.J., Lamiquiz-Moneo I., Lasarte-Sanz I., Sala-Fernández L., Marín-Andrés M., Rubio-Sánchez P., Moneo-Hernández M.I., Hernández-Aguilar M.T. (2023). Prevalence of overweight and obesity in Aragón and variations according to health determinants. An. Pediatr..

[5] Ng M., Fleming T., Robinson M., Thomson B., Graetz N., Margono C., Mullany E.C., Biryukov S., Abbafati C., Abera S.F. (2014). Global, regional, and national prevalence of overweight and obesity in children and adults during 1980–2013: A systematic analysis for the Global Burden of Disease Study 2013. Lancet.

[6] Tamborlane Editor W.V. Contemporary Endocrinology Series Editor: Leonid Poretsky Diabetes in Children and Adolescents A Guide to Diagnosis and Management [Internet]. http://www.springer.com/.

[7] Chou Y.H., Su Y.T., Lo F.S., Chiu C.F., Huang Y.C. (2024). Influencing factors for treatment escalation from metformin monotherapy in youth-onset type 2 diabetes in Northern Taiwan. Pediatr Neonatol..

[8] Siller A.F., Tosur M., Relan S., Astudillo M., McKay S., Dabelea D., Redondo M.J. (2020). Challenges in the diagnosis of diabetes type in pediatrics. Pediatric Diabetes.

[9] Tamborlane W., Shehadeh N. (2023). Unmet Needs in the Treatment of Childhood Type 2 Diabetes: A Narrative Review. Adv. Ther..

[10] Or T., Lm T., Mr P. (2016). Type 2 diabetes mellitus in children and adolescents: A relatively new clinical problem within pediatric practice. J. Med. Life.

[11] Newton K.P., Wilson L.A., Crimmins N.A., Fishbein M.H., Molleston J.P., Xanthakos S.A., Behling C., Schwimmer J.B., Garner D., Hertel P. (2023). Incidence of Type 2 Diabetes in Children With Nonalcoholic Fatty Liver Disease. Clin. Gastroenterol. Hepatol..

[12] Barrett T., Jalaludin M.Y., Turan S., Hafez M., Shehadeh N. (2020). Rapid progression of type 2 diabetes and related complications in children and young people—A literature review. Pediatr. Diabetes.

[13] Popoviciu M.S., Kaka N., Sethi Y., Patel N., Chopra H., Cavalu S. (2023). Type 1 Diabetes Mellitus and Autoimmune Diseases: A Critical Review of the Association and the Application of Personalized Medicine. J. Pers. Med..

[14] Huerta-Uribe N., Hormazábal-Aguayo I.A., Izquierdo M., García-Hermoso A. (2023). Youth with type 1 diabetes mellitus are more inactive and sedentary than apparently healthy peers: A systematic review and meta-analysis. Diabetes Research and Clinical Practice.

[15] Alfaraidi H., Samaan M.C. (2023). Metformin therapy in pediatric type 2 diabetes mellitus and its comorbidities: A review. Front. Endocrinol..

[16] Axon E., Atkinson G., Richter B., Metzendorf M.I., Baur L., Finer N., Corpeleijn E., O’Malley C., Ells L.J., Cochrane Metabolic Endocrine Disorders Group (2016). Drug interventions for the treatment of obesity in children and adolescents. Cochrane Database of Systematic Reviews.

[17] Sikorskaya K., Zarzecka I., Ejikeme U., Russell J. (2021). The use of metformin as an add-on therapy to insulin in the treatment of poorly controlled type 1 diabetes mellitus in adolescents. Metabol. Open..

[18] Roep B.O., Thomaidou S., van Tienhoven R., Zaldumbide A. (2021). Type 1 diabetes mellitus as a disease of the β-cell (do not blame the immune system?). Nature Reviews Endocrinology. Nat. Res..

[19] Satterwhite L.E. (2021). Metformin Extended-Release Oral Solution. Clin. Diabetes.

[20] Racaniello G.F., Silvestri T., Pistone M., D’Amico V., Arduino I., Denora N., Lopedota A.A. (2024). Innovative Pharmaceutical Techniques for Paediatric Dosage Forms: A Systematic Review on 3D Printing, Prilling/Vibration and Microfluidic Platform. J. Pharm..

[21] Lajoinie A., Janiaud P., Henin E., Gleize J.C., Berlion C., Nguyen K.A., Nony P., Gueyffier F., Maucort-Boulch D., Koupaï B.K. (2017). Assessing the effects of solid versus liquid dosage forms of oral medications on adherence and acceptability in children. Cochrane Database Syst. Rev..

[22] Bryson S.P. (2014). Patient-centred, administration friendly medicines for children—An evaluation of children’s preferences and how they impact medication adherence. Int. J. Pharm..

[23] Venables R., Batchelor H., Hodson J., Stirling H., Marriott J. (2015). Determination of formulation factors that affect oral medicines acceptability in a domiciliary paediatric population. Int. J. Pharm..

[24] Mistry P., Batchelor H. (2017). Evidence of acceptability of oral paediatric medicines: A review. J. Pharm. Pharmacol..

[25] Meyers R.S. (2024). The Past, Present, and Future of Oral Dosage Forms for Children. J. Pediatr. Pharmacol. Ther..

[26] Cram A., Breitkreutz J., Desset-Brèthes S., Nunn T., Tuleu C. (2009). Challenges of developing palatable oral paediatric formulations. Int. J. Pharm..

[27] Cui M., Pan H., Su Y., Fang D., Qiao S., Ding P., Pan W. (2021). Opportunities and challenges of three-dimensional printing technology in pharmaceutical formulation development. Acta Pharm. Sin. B.

[28] Malebari A.M., Kara A., Khayyat A.N., Mohammad K.A., Serrano D.R. (2022). Development of Advanced 3D-Printed Solid Dosage Pediatric Formulations for HIV Treatment. Pharmaceuticals.

[29] Goyanes A., Scarpa M., Kamlow M., Gaisford S., Basit A.W., Orlu M. (2017). Patient acceptability of 3D printed medicines. Int. J. Pharm..

[30] Anaya B.J., Cerda J.R., D’Atri R.M., Yuste I., Luciano F.C., Kara A., Ruiz H.K., Ballesteros M.P., Serrano D.R. (2023). Engineering of 3D printed personalized polypills for the treatment of the metabolic syndrome. Int. J. Pharm..

[31] Rouaz-El Hajoui K., Herrada-Manchón H., Rodríguez-González D., Fernández M.A., Aguilar E., Suné-Pou M., Nardi-Ricart A., Pérez-Lozano P., García-Montoya E. (2023). Pellets and gummies: Seeking a 3D printed gastro-resistant omeprazole dosage for paediatric administration. Int. J. Pharm..

[32] Lee J., Song C., Noh I., Rhee Y.S. (2024). Applications of the design of additive manufacturing (DfAM) in the development of pharmaceutical dosage forms. J. Pharm. Investig..

[33] Ganatra P., Jyothish L., Mahankal V., Sawant T., Dandekar P., Jain R. (2024). Drug-loaded vegan gummies for personalized dosing of simethicone: A feasibility study of semi-solid extrusion-based 3D printing of pectin-based low-calorie drug gummies. Int. J. Pharm..

[34] Herrada-Manchon H., Rodriguez-Gonzalez D., Alejandro Fernandez M., Sune-Pou M., Perez-Lozano P., Garcia-Montoya E., Aguilar E. (2020). 3D printed gummies: Personalized drug dosage in a safe and appealing way. Int. J. Pharm..

[35] Tagami T., Ito E., Kida R., Hirose K., Noda T., Ozeki T. (2021). 3D printing of gummy drug formulations composed of gelatin and an HPMC-based hydrogel for pediatric use. Int. J. Pharm..

[36] Serrano D.R., Kara A., Yuste I., Luciano F.C., Ongoren B., Anaya B.J., Molina G., Diez L., Ramirez B.I., Ramirez I.O. (2023). 3D Printing Technologies in Personalized Medicine, Nanomedicines, and Biopharmaceuticals. Pharmaceutics.

[37] Mohammed A., Elshaer A., Sareh P., Elsayed M., Hassanin H. (2020). Additive Manufacturing Technologies for Drug Delivery Applications. Int. J. Pharm..

[38] Ngo T.D., Kashani A., Imbalzano G., Nguyen K.T.Q., Hui D. (2018). Additive manufacturing (3D printing): A review of materials, methods, applications and challenges. Compos. Part B Eng..

[39] Durga Prasad Reddy R., Sharma V. (2020). Additive manufacturing in drug delivery applications: A review. Int. J. Pharm..

[40] Xu X., Awad A., Robles-Martinez P., Gaisford S., Goyanes A., Basit A.W. (2021). Vat photopolymerization 3D printing for advanced drug delivery and medical device applications. J. Control. Release.

[41] Rodríguez-Pombo L., de Castro-López M.J., Sánchez-Pintos P., Giraldez-Montero J.M., Januskaite P., Duran-Piñeiro G., Bóveda M.D., Alvarez-Lorenzo C., Basit A.W., Goyanes A. (2024). Paediatric clinical study of 3D printed personalised medicines for rare metabolic disorders. Int. J. Pharm..

[42] Zhu C., Tian Y., Zhang E., Gao X., Zhang H., Liu N., Han X., Sun Y., Wang Z., Zheng A. (2022). Semisolid Extrusion 3D Printing of Propranolol Hydrochloride Gummy Chewable Tablets: An Innovative Approach to Prepare Personalized Medicine for Pediatrics. AAPS PharmSciTech.

[43] Seoane-Viaño I., Januskaite P., Alvarez-Lorenzo C., Basit A.W., Goyanes A. (2021). Semi-solid extrusion 3D printing in drug delivery and biomedicine: Personalised solutions for healthcare challenges. J. Control. Release.

[44] Díaz-Torres E., Rodríguez-Pombo L., Ong J.J., Basit A.W., Santoveña-Estévez A., Fariña J.B., Alvarez-Lorenzo C., Goyanes A. (2022). Integrating pressure sensor control into semi-solid extrusion 3D printing to optimize medicine manufacturing. Int. J. Pharm. X.

[45] Junnila A., Mortier L., Arbiol A., Harju E., Tomberg T., Hirvonen J., Viitala T., Karttunen A.P., Peltonen L. (2024). Rheological insights into 3D printing of drug products: Drug nanocrystal-poloxamer gels for semisolid extrusion. Int. J. Pharm..

[46] Tong H., Zhang J., Ma J., Zhang J. (2024). Perspectives on 3D printed personalized medicines for pediatrics. Int. J. Pharm..

[47] Chimene D., Lennox K.K., Kaunas R.R., Gaharwar A.K. (2016). Advanced Bioinks for 3D Printing: A Materials Science Perspective. Ann. Biomed. Eng..

[48] Lille M., Nurmela A., Nordlund E., Metsä-Kortelainen S., Sozer N. (2018). Applicability of protein and fiber-rich food materials in extrusion-based 3D printing. J. Food Eng..

[49] Niu D., Zhang M., Tang T., Mujumdar A.S., Li J. (2023). Investigation of 3D printing of children starch gummies with precise and special shape based on change of model parameters. J. Food Eng..

[50] Wang R., Hartel R.W. (2021). Confectionery gels: Gelling behavior and gel properties of gelatin in 1 concentrated sugar solutions. Food Hydrocoll..

[51] Agencia Española de Medicamentos y Productos Sanitarios (2023). FICHA TECNICA METFORMINA STADA 850 mg COMPRIMIDOS RECUBIERTOS CON PELICULA EFG [Internet]. https://cima.aemps.es/cima/dochtml/ft/69709/FichaTecnica_69709.html.

[52] Rong L., Chen X., Shen M., Yang J., Qi X., Li Y., Xie J. (2023). The application of 3D printing technology on starch-based product: A review. Trends Food Sci. Technol..

[53] Serrano D.R., Walsh D., O’Connell P., Mugheirbi N.A., Worku Z.A., Bolas-Fernandez F., Galiana C., Dea-Ayuela M.A., Healy A.M. (2018). Optimising the in vitro and in vivo performance of oral cocrystal formulations via spray coating. Eur. J. Pharm. Biopharm..

[54] U.S.P. Reagents: Test Solutions. https://www.uspnf.com/sites/default/files/usp_pdf/EN/USPNF/pf-legacy-pdf/pf-2015_vol-41.pdf.

[55] Galana Gerlin M.C., Mazon Cardoso T.F., Souza J.B.G.D., Baroni A.C.D.M., Amaral M.S.D., Kassab N.M. (2022). Desenvolvimento e validação de método analítico por CLAE para determinação simultânea de atorvastatina, losartana e metformina em formulações farmacêuticas magistrais. Rev. Colomb. Cienc. Quím. Farm..

